# Drooling outcome measures in paediatric disability: a systematic review

**DOI:** 10.1007/s00431-022-04460-5

**Published:** 2022-04-20

**Authors:** E. Sforza, R. Onesimo, C. Leoni, V. Giorgio, F. Proli, F. Notaro, E. M. Kuczynska, A. Cerchiari, A. Selicorni, D. Rigante, G. Zampino

**Affiliations:** 1 Centre for Rare Diseases and Birth Defects, Department of Woman and Child Health and Public Health, Fondazione Policlinico Universitario A. Gemelli IRCCS, Largo A. Gemelli 8, 00168 Rome, Italy; 2 Feeding and Swallowing Services Unit, Department of Neuroscience and Neurorehabilitation, Bambino Gesù Children’s Hospital IRCCS, Rome, Italy; 3 Department of Paediatrics, ASST Lariana, Presidio S. Fermo, Como, Italy; 4 Department of Pediatrics, Faculty of Medicine and Surgery, Catholic University of Rome, Rome, Italy

**Keywords:** Drooling, Sialorrhea, Disability, Paediatrics, Personalised medicine, Systematic review

## Abstract

Drooling, or sialorrhea, is a common condition in patients with cerebral palsy, rare diseases, and neurodevelopmental disorders. The goal of this review was to identify the different properties of sialorrhea outcome measures in children. Four databases were analysed in search of sialorrhea measurement tools, and the review was performed according to the Preferred Reporting Items for Systematic reviews and Meta-Analyses (PRISMA) statement. The COnsensus-based Standards for the selection of health status Measurement INstruments (COSMIN) checklist was used for quality appraisal of the outcome measures. The initial search yielded 891 articles, 430 of which were duplicates. Thus, 461 full-text articles were evaluated. Among these, 21 met the inclusion criteria, reporting 19 different outcome measures that encompassed both quantitative measures and parent/proxy questionnaires.

*   Conclusions*: Among the outcome measures found through this review, the 5-min Drooling Quotient can objectively discriminate sialorrhea frequency in patients with developmental disabilities. The Drooling Impact Scale can be used to evaluate changes after treatment. The modified drooling questionnaire can measure sialorrhea severity and its social acceptability. To date, the tests proposed in this review are the only tools displaying adequate measurement properties. The acquisition of new data about reliability, validity, and responsiveness of these tests will confirm our findings.

**Table:** 

**What is Known:** *•* *Although sialorrhea is a recognized problem in children with disabilities, especially those with cerebral palsy (CP), there is a lack of confidence among physicians in measuring sialorrhea.*	
**What is New:** *• Few sialorrhea measures are available for clinicians that may guide decision-making and at the same time have strong evidence to provide confidence in the results.* *•* *A combination of both quantitative measures and parent/proxy questionnaires might provide an adequate measurement of sialorrhea in children.*

**Supplementary information:**

The online version contains supplementary material available at 10.1007/s00431-022-04460-5.

## Introduction

Drooling, or sialorrhea, is a well-recognised health issue in children with disabilities, especially those with cerebral palsy (CP). It can be defined as the unintentional spill of saliva from the mouth [1], even if several other definitions have been reported [2–6]. Although sialorrhea is normal in infants, it is considered pathological after the age of 4 years old [7]. In addition, severe sialorrhea can give rise to a number of limiting physical and psychosocial complications such as social isolation and low self-esteem [1, 8].

Although sialorrhea severity varies daily, and sometimes hourly or depending on daily life circumstances, there is a need to quantify its frequency and its impact on children’s and their caregivers’ quality of life [9]. Various interventions have been described to reduce or eliminate sialorrhea. These include surgery, botulinum toxin (BoNT-A and BoNT-B), anticholinergic medications, and oral-motor therapies [1]. This challenging condition should always be addressed by a multidisciplinary team, specifically by professionals with experience in disability and in children with special needs [10]. However, there currently is a lack of knowledge among paediatricians on how to adequately quantify sialorrhea. In fact, Parr et al. found that very few paediatricians in the UK use standardised methods to measure sialorrhea and the effectiveness of medications or their adverse effects [9]. Hence, the aim of this review was to appraise the measurement properties of drooling measures validated in the paediatric population.

## Methods

### Search strategy

Supervised by R.O., E.S. performed a systematic electronic literature search of the following databases: PubMed, Scopus, Cochrane Library, and CINAHL (EBSCO). Search terms combined text words and Medical Subject Headings (MeSH), as shown in Supplementary Table 1. MeSH terms included three components: terms referring to drooling/sialorrhea, target population and assessment methods.

### Study eligibility

Following the Preferred Reporting Items for Systematic reviews and Meta-Analyses (PRISMA) checklist [11] (Supplementary Table 2) and after removing duplicates, all full-text articles were screened by two independent researchers; any discrepancies were solved in a consensus meeting. The articles were included if they reported objective or subjective outcome measures of sialorrhea that were appropriate for use in children aged 0–18 years with or without special needs, that were freely-available, and written in English. No date limit was set, to avoid excluding potentially useful evaluation methods and questionnaires. Exclusion criteria were absence of statistical numerical results within the study except for those studies describing an outcome measure for the first time, those only assessing salivary production and those evaluating post-therapeutic outcomes.

### Data collection and assessment

Included studies were assessed independently by two researchers. Sialorrhea outcome measures identified in all selected papers were classified depending on two domains: quantitative measures *versus* parent or proxy reports with quality of life evaluation. Articles were reviewed for the evaluation of qualitative features, such as domain assessed, time needed for questionnaire administration, population, and age of population. Scoring and its interpretation were also extracted. If the article was deemed worthy of inclusion but was lacking specific information, its corresponding author could be contacted for clarifications.

The COnsensus-based Standards for the selection of health status Measurement INstruments (COSMIN) checklist (July 2019 version) [12] was used to evaluate the methodological quality of each outcome measure described in the included studies. The COSMIN checklist was developed by authors based on previous COSMIN checklist versions [13, 14] and on the COSMIN Risk of Bias checklist for PROMs [15, 16]. A 4-point rating scale (very good, adequate, doubtful, inadequate) was used to assess each standard recommended by the checklist in each article. As the COSMIN checklist does not provide an overall rating score, we used the “worst-score counts principle” [14] to obtain one.

Data on validity, reliability, and responsiveness (described in Supplementary Table 3) of all measures were also collected, though data collection on construct validity, content validity, and internal consistency was not applicable for quantitative outcome measures. In addition, the quantitative results for each study have been rated against the Terwee et al. [17] criteria.

A positive rating was assigned to sensitivity and specificity when equal or over 0.80 [18], to criterion validity if the correlation with the gold standard was at least 0.70 [17], to reliability when the intraclass correlation coefficient (ICC) or weighted Kappa was at least 0.70 in a sample size of at least 50 patients [17], and to measurement error if authors provided convincing arguments that it was acceptable. A positive rating was given to internal consistency when factor analysis was applied and Cronbach’s alpha was between 0.70 and 0.95 [17]. For responsiveness, the area under the receiver operating characteristic (ROC) curve (AUC) of at least 0.70 or Guyatt’s responsiveness ratio (RR) of at least 1.96 was considered adequate [17]. A gold standard for measuring sialorrhea was considered “gold” only when it was the original long version to which a shortened instrument was compared to. Feasibility was rated as adequate if the test needed up to 10–15 min to be completed and if the questionnaire was self-administered [18]. The primary purpose (predictive, discriminative, or evaluative) of tools evaluating sialorrhea was also assessed [17, 19].

## Results

**Fig. 1:**
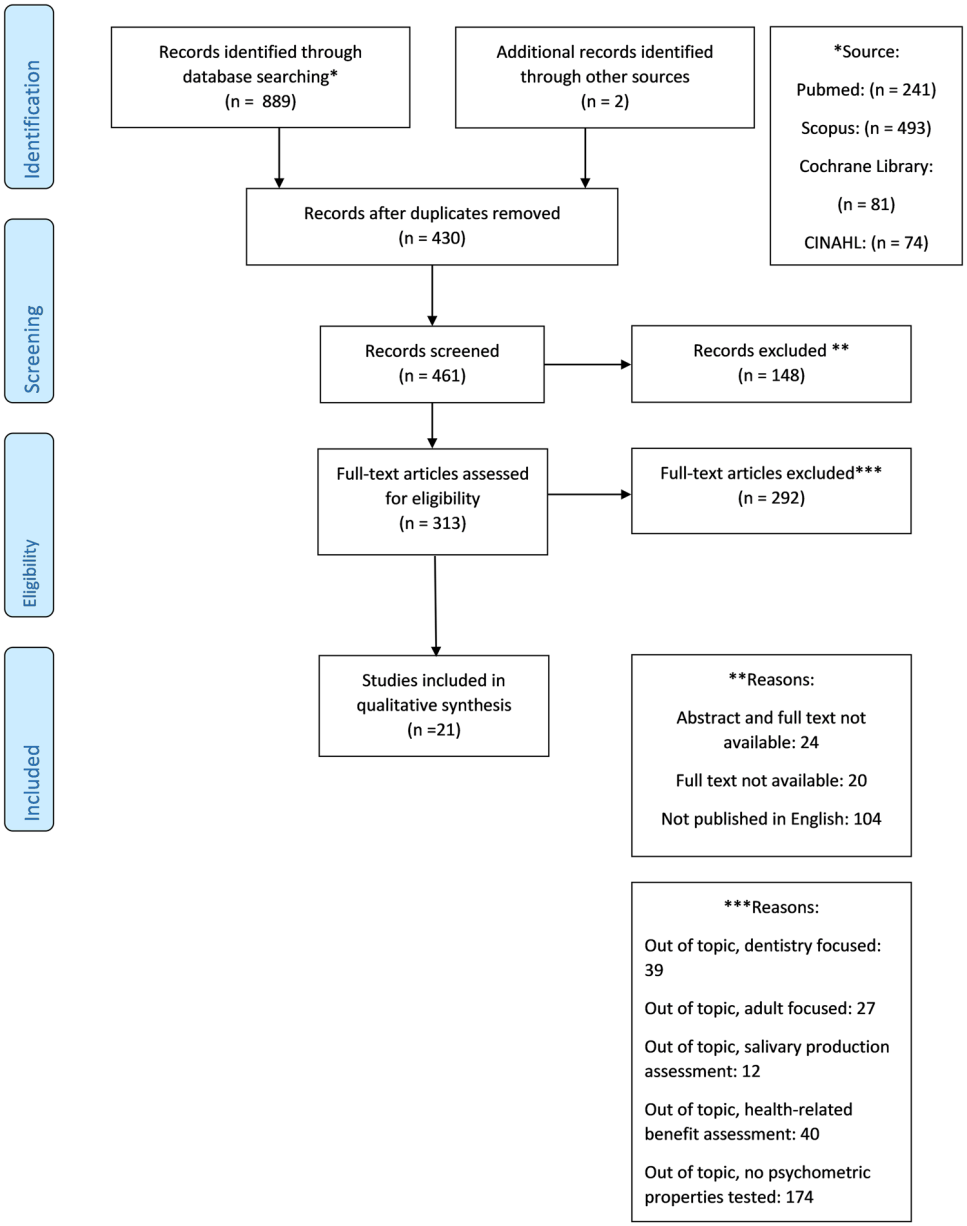
Diagram of literature search and article selection

The initial literature search yielded 891 articles. Duplicates (*n* = 430) were excluded and the remaining 461 “full-text” manuscripts were evaluated**.** Agreement between the two independent researchers reviewing the articles was high (Cohen’s Kappa > 0.8). Of the 461, 21 studies met the inclusion criteria, as shown in Fig. 1. Only one author (van der Burg) [8] had to be contacted to clarify the exact number of questionnaires developed in his study. Overall, 19 sialorrhea assessment tools were identified (Table 1): 5 quantitative/semi-quantitative outcome measures [20–24] and 14 questionnaires measuring severity and/or impact of sialorrhea on patients’ quality of life [1, 6, 8, 25–35]. Extensive description and explanation of each tool can be found in the Supplementary material.

**Table 1:** Measures of sialorrhea

Type of measure	Name of measure
**Quantitative/semiquantitative outcome methods**	Bib count [20] Bib weight [21] Sochaniwskyj’s technique [22] Drooling Quotient [23] 5-min Drooling Quotient (DQ5) [24]
**Scales and questionnaires measuring severity**	Drooling Infants and Preschoolers Scale (DRIPS) [25] Drooling Severity and Frequency Scale (DSFS) [26] Blasco Index for the assessment of drooling [1] Teacher Drool Scale (TDS) [27] Modified Teacher Drool Scale (mTDS) [28] Visual Analogue Scale (VAS) [29]
**Scales and questionnaires measuring severity, impact on quality of life and daily life**	Modified drooling questionnaire [30] Drooling Impact Scale (DIS) [31] French version of Drooling Impact Scale (DIS-F) [32] Brazilian Portuguese language version of DIS [33] Drooling impact questionnaire (short version) [6] Questionnaire to evaluate impact of drooling on daily living (questionnaire 1; questionnaire 2) [8] Daniel Drooling Impact Score Questionnaire (DDISQ) [34] Drool rating scale [35]

### Qualitative and quantitative features

The assessment tools differed in sialorrhea quantification methodology and purpose of assessment.

Sialorrhea was quantified by counting bibs changed daily [20], weighing bibs [21], collecting saliva with a cup [22], direct/standardised observation of sialorrhea episodes over a 5 to 10-min period [23, 24], and through subjective scales or questionnaires [1, 6, 8, 25–35].

The primary purpose of assessment was predictive for bib count [20] and bib weight [21], discriminative for the Modified drooling questionnaire [30], the 5-min Drooling Quotient (DQ5) [24], the Drooling Infants and Preschoolers Scale (DRIPS) [25], and the Blasco Index for the assessment of drooling [1], evaluative for all the others [6, 8, 22, 23, 26–29, 31, 33–35].

Responsiveness data were available only for the Drooling Impact Scale (DIS) [31] and the French version of the Drooling Impact Scale (DIS-F) [32], while other measures had been used in several clinical trials to measure longitudinal changes in sialorrhea after treatment. Data related to target population, sample size, and feasibility are listed in Table 2.

**Table 2:** Outcome measures, structure, and scoring

**Outcome measure**	**Target population; age; gender**	**Study population**	**Procedure; administration time; clear instruction; manual**	**Cut-off and interpretation of scores**
**Bib count** [20, 38]	Developmental disabilities; 6 m–18y; 241 M, 173F	414	Bib counting; 1 day; yes; NA	NR
	Children with neurological disorder and drooling; 4–18y; 81 M, 74F	155	Bib counting; 1 day; yes; NA	NR
**Bib** **weight** [21]	Children with developmental disabilities;8–18y; -	14	Bib weighing; 10 min; yes; NA; NA	NR
**Sochaniwskyj's Technique** [22]	NR	NR	Collecting saliva leaking from the mouth with a cup; 30 min × 5 time in a day; yes; NA	NR
**DQ5** [24]	Developmental disabilities and moderate/profuse drooling; 4–22y; 101 M 61F	162	Observation of drooling episodes; 5 min; yes; NA	A cut-off of 18 or more might indicate ‘constant drooling’
**DQ** [23, 38]	Children with CP and normally developed children; 10–16y; NR	24	Observation of drooling episodes; 10 min yes; NA	A higher value represents a worse outcome
	Children with neurological disorder and drooling; 4–18y; 81 M, 74F	155	Observation of drooling episodes; 10 min yes; NA	> 97th percentile: pathological; > 85th percentile: at risk
**DRIPS** [25]	Typically developing children; 0–4y; 314 M, 338F	652	Observational, parent report; 15 min; yes; no	Combined subscales rankings Tot score: from 2 to 9 A higher value represents a worse outcome; in case of high value on all factors it is suggested that an overall developmental delay may be an underlying cause
**DSFS** [26, 38]	Typically developed and children with developmental disabilities; 2–23y	36	Observational, investigator and parent report; NR; yes; no	A higher value represents a worse outcome
	Children with neurological disorder and drooling; 4–18y; 81 M, 74F	155	Observational, parent report; NR; yes; no	A higher value represents a worse outcome
**Balsco index for the assessment of drooling **[1]	NR	NR	NR; NR; NR; no	A higher value represents a worse outcome
**TDS** [37]	CP; 4–44y; 11 M, 9F	20	Observational, teacher report; Full school day observation; NR; no	A higher value represents a worse outcome
**mTDS** [28]	Neurodevelopmental conditions and severe drooling; 4–19y; NR	39	Observational, parent report; NR; yes; no	A higher value represents a worse outcome
**VAS** [29]	CP; 3–17y; 28 M, 17F	45	Observational, investigator and parent report; NR; NR; no	A score of 24 is a cut-off between the dry and mild, and the moderate and severe droolers
**Modified drooling questionnaire** [30]	Children with CP and drooling; 4–16 y; 72 M, 42F	113	Investigator administration; 10 min; yes; no	The total score is reported and is calculated by adding the score of all 10 subscales. A higher value represents a worse outcome
**DIS** [31]	Developmental disabilities; 4–18 y; 51 M, 29F	stable group (*n* = 31) and intervention group (*n* = 49)	Observational, parent report; NR; yes; no	The total score is reported and is calculated by adding the score of all 10 subscales. A higher value represents a worse outcome
**DIS-F** [32]	Children with CP and drooling, 4–18 y; 32 M, 23F	Control group (*n* = 33), intervention group (*n* = 22)	Observational, parent report; NR; yes; no	The total score is reported and is calculated by adding the score of all 10 subscales. A higher value represents a worse outcome
**Brazilian Portuguese language version of DIS** [33]	Children or adolescent with drooling 19.75 − 150.75 months; 20 M, 20F,	40	Observational, parent report; NR; yes; no	The total score is reported and is calculated by adding the score of all 10 subscales. A higher value represents a worse outcome
**Drooling impact** **questionnaire** **(short** **version)** [6, 36]	Children or adolescent with drooling, 7–19y; 5 M, 5F	10	Observational, parent report; NR; yes; no	NR
	Children with CP and severe drooling, 3–16 y; 28 M, 17F	45	Observational, parent report; NR; yes; no	NR
**Questionnaire to evaluate impact of drooling on daily living (questionnaire 1)** [8]	Children with CP and severe drooling; 3–16 y; 28 M, 17F	45	Observational, parent report; NR; yes; no	NR
**Questionnaire to evaluate impact of drooling on daily living (questionnaire 2)** [8]	Children with CP and severe drooling; 3–16 y; 28 M, 17F	45	Observational, parent report; NR; yes; no	NR
**DDISQ** [34]	NR	NR	Observational, parent report; NR; yes; no	NR
**Drool** **rating** **scale** [35]	Children with CP and drooling; 8–21 y; NR	22	Observational, parent report; NR; yes; no	A higher value represents a worse outcome

*CP* cerebral palsy, *DDISQ* Daniel Drooling Impact Score Questionnaire, *DIS* Drooling Impact Scale, *DIS-F* French version of Drooling Impact Scale, *DQ* Drooling Quotient, *DQ5* 5-min Drooling Quotient, *DQ5A* 5-min Drooling Quotient during activities, *DQ5R* 5-min Drooling Quotient at rest, *DRIPS* Drooling Infants and Preschoolers Scale, *DSFS* Drooling Severity and Frequency Scale, *F* female, *M* male, *NA* not applicable, *NR* not reported, *QoL* quality of life, *TDS* Teacher Drooling Scale, *VAS* Visual Analogue Scale, *Y* years

Among scales and questionnaires, there was an adequate feasibility for the DRIPS [25], which is self-administered and performed in 15 min, and for the Modified drooling questionnaire [30] that needs a mean administration time of 10 min. Administration time was reported also for the Teacher Drooling Scale (TDS) [27], requiring a full school day observation.

### Validity and reliability

With regard to measurement properties, data for both reliability and validity were available for the DQ5 [24], the modified drooling questionnaire [30], the DIS [31], the French version of Drooling Impact Scale (DIS-F) [32], the Brazilian Portuguese language version of DIS [33], the TDS [27], the DQ [23], and the DRIPS [25]. The Drooling Impact Questionnaire (short version) [6, 36], the questionnaire to evaluate impact of drooling on daily living (questionnaires 1 and 2) [8], bib count [20], bib weight [21], the Drooling Severity and Frequency Scale (DSFS) [26], the Visual Analogue Scale (VAS) [29], and the Daniel Drooling Impact Score Questionnaire (DDISQ) [34], reported only data on validity. The remaining outcome measures did not have any measurement properties tested [1, 22, 28, 35]. The different aspects of reliability (i.e. Inter-rater, Intra-rater, test retest) and validity available for each sialorrhea measure are shown in Tables 3 and 4.

**Table 3:** Outcome measures, validity and responsiveness

**Outcome measure**	**Content validity**	Construct validity*	**Concurrent validity**	**Predictive validity**	**Sensitivity and specificity**	**Responsiveness**
**Bib count** [20, 38]	NA	NA	Pearson *r* = 0.416, *p* < 0.01 correlated with drooling frequency of DDISQ **(-)** *r* = 0.541 Pearson *p* < 0.01 correlated with drooling severity of DDISQ **(-)**	*β* = 1.14, *p* = 0.001 for severity **(+);** *β* = 0.25, *p* = 0.058 for frequency **(-)**	NR	NR
	NA	NA	Correlated with DQ scale Spearman’s ρ 0.227 (*p* = 0.005) **(-)**; correlated with DSFS Spearman’s ρ 0.335 (*p* = < 0.001) **(-)**	NR	NR	NR
**Bib weight **[21]	NA	NA	Spearman’s ρ 0.604, *p* < 0,05 correlated with cumulative drooling quotient of DSFS **(-)**	NR	NR	NR
**Sochaniwskyj’s Technique **[22]	NA	NA	NR	NR	NR	NR
**DQ5 **[24]	NA	NA	ICC > 0,9 between DQ10A and DQ5A **(+)**; ICC > 0,9 between DQ10R and DQ5R **(+)**; Pearson’s *r* between VAS and DQ5A 0.45 (0.32–0.58) **(-)**; Pearson’s *r* between VAS and DQ5R 0.45 (0.21–0.49) **(-)**	NR	DQ5A sensitivity of 0.61 and specificity of 0.75 with a cut-off of 18, AUC 0.80 (0.73–0.88) **(+)**; DQ5R sensitivity of 0.45 and specificity of 0.87 with a cut-off of 18, AUC 0.69 (0.60–0.78) **(-)**	NA
**DQ** [23, 38, 30]	NA	NA	NR	NR	NR	NR
	NA	NA	Correlated with DSFS in neurological disorders (*n* = 62) Spearman’s ρ 0.900 *p* < 0.001 **(+)**; correlated with DSFS in developmental delay (*n* = 64) Spearman’s ρ 0.888 *p* < 0.001 **(+)**; correlated with bib count in neurological disorders (*n* = 62) Spearman’s ρ 0.271 *p* = 0.032 **(-)**; correlated with bib count in developmental delay (*n* = 64) Spearman’s ρ 0.155 p = 0.2200 **(-)**; correlated with DS of DSFS in neurological disorders (*n* = 62) Spearman’s ρ 0.893 *p* < 0.001 **(+)**; correlated with DS of DSFS in developmental delay (*n* = 64) Spearman’s ρ 0.887 *p* < 0.001 **(+)**; correlated with DF of DSFS in neurological disorders (*n* = 62) Spearman’s ρ 0.659 *p* < 0.001 **(-)**; correlated with DF of DSFS in developmental delay (*n* = 64) Spearman’s ρ 0.690 *p* < 0.001 **(-)**	NR	NR	NR
	NA	NA	Correlated with modified drooling questionnaire 0.83 to 0.87 *p* < 0.001 **(+)**	NA	NR	NR
**DRIPS **[25]	Item generation based on common knowledge about drooling, children’s psychomotor development, and the development of saliva control	PCA conducted on 20 items (+)	NA	NR	NA	NR
**DSFS **[26, 38, 21]	NR	NR	NR	NR	NR	NR
	NR	NR	DSFS tot correlated with DQ scale Spearman’s ρ 0.886 (*p* < 0.001) **(+)**; DSFS tot correlated with Bib Changes Spearman’s ρ 0.335 (*p* < 0.001) **(-)**. DSS correlated with DQ scale Spearman’s ρ 0.898 (*p* < 0.001) **(+)**; DFS correlated with DQ scale Spearman’s ρ 0.653 (*p* < 0.001) **(-)**;	NR	NR	NR
	NR	NR	Spearman rho 0.604, *p* < 0,05 correlated with bib weight **(-)**	NR	NR	NR
**Balsco index for the assessment of drooling **[1]	NR	NR	NR	NR	NR	NR
**TDS** [27]	NR	NR	TDS correlated with time-sampling tot ρ 0.665 (*p* < 0.01) **(-)**; TDS correlated with time-sampling stream ρ 0.881 (*p* < 0.001) **(+)**	NR	NR	NR
**mTDS **[28]	NR	NR	NR	NR	NR	NR
**VAS **[29, 24]	NA	NA	NR	NR	NR	NR
	NA	NA	Pearson’s *r* between VAS and DQ5A 0.45 (0.32–0.58) **(-)**; Pearson’s* r* between VAS and DQ5R 0.45 (0.21–0.49) **(-)**	NR	NR	NR
**Modified drooling questionnaire **[30]	Item generation based on existing questionnaires adapted to the local context	Cross-cultural validity	Correlated with DQ 0.83 to 0.87 *p* < 0.001 **(+)**	ROC area 0.9417 (95% CI 0.88 to 0.99) **(+)**	NR	NR
**DIS **[31]	Item generation gained from parents and expert opinion of speech pathologists	Correlated with carer’s global rating of change in drooling 0.69 *p* < 0.001 **(+)**	NR	NR	NR	RR 1.4 **(-)**; mean change in stable group 0; difference between groups 23.5 *p* < 0.001
**DIS-F **[32]	Items translated according to Beaton et al. guidelines [39]	Cross-cultural validity	NR	NR	NR	difference between groups 36.5 [95% CI = 26.4; 46.6 (*p* < 0.0001)
**Brazilian Portuguese language version of DIS** [33]	Items translated according to Beaton et al. guidelines [39]	Cross-cultural validity	NR	NR	NR	NR
**Drooling impact questionnaire (short version)** [6, 36]	NR	NR	NR	NR	NR	NR
	Items selected by interdisciplinary team in accordance with parents’ opinion	NR	NR	NR	NR	NR
**Questionnaire to evaluate impact of drooling on daily living (questionnaire 1 and questionnaire 2) **[8]	Team reached consensus on selected items regarding whether they reflected relevant aspects of the impact of drooling on daily life by expert team	NR	NR	NR	NR	NR
**DDISQ** [34, 20]	NR	NR	NR	NR	NR	NR
	NR	NR	Drooling frequency of DDISQ correlated with Bib count Pearson* r* = 0.416, *p* < 0.01 **(-)**; drooling severity of DDISQ correlated with Bib count Pearson *r* = 0.541, *p* < 0.01 **(-)**	NR	NR	NR
**Drool rating scale **[35]	NR	NR	NR	NR	NR	NR

*AUC* area under curve, *β* standardised beta, *p* probability value, *DDISQ* Daniel Drooling Impact Score Questionnaire, *DIS* Drooling Impact Scale, *DIS-F* French version of Drooling Impact Scale, *DQ* Drooling Quotient, *DQ5* 5-min Drooling Quotient, *DQ5A* 5-min Drooling Quotient during activities, *DQ5R* 5-min Drooling Quotient at rest, *DRIPS* Drooling Infants and Preschoolers Scale, *DSFS* Drooling Severity and Frequency Scale, *NA* not applicable, *PCA* principal component analysis, *RR* responsiveness ratio, *ROC* receiver operating characteristics, *TDS* Teacher Drooling Scale, *VAS* Visual Analogue Scale, (+) positive rating, (-) negative rating

^*^(structural validity, hypotheses-testing, cross-cultural validity)

**Table 4:** Outcome measures, reliability

**Outcome measure**	**Inter-rater reliability and measurement error**	**Independent administration and similar test condition**	**Intra-rater reliability and measurement error**	**Appropriate time interval**	**Test–retest reliability and measurement error**	**Internal consistency**
**Bib count **[20]	NR	NR	NR	NR	NR	NA
**Bib weight **[21]	NR	NR	NR	NR	NR	NA
**Sochaniwskyj's Technique **[22]	NA	NA	NR	NR	NR	NA
**DQ5 **[24]	4 observers: ICC 0.91 (95% CI 0.67–0.98) **(+)**, ICC 0.86 (95% CI 0.55–0.96) **(+)**, ICC 0.95 (95% CI 0.80–0.99) **(+),** ICC 0.91(95% C I0.67–0.98) **(+)**. Small systematic error between DQ10A and DQ5A scores (0.2; SD 6.39); limits of agreement between DQ10 and DQ5 10%, acceptable random error; systematic error between DQ5A and DQ5R 5.74 (SD 16.5) **(+)**	Yes	ICC 0.95 (95% CI 0.85–0.99) **(+)**	Yes	NR	NA
**DQ **[23]	99% agreement measured on one patient **(-)**	Yes	NR	NR	NR	NA
**DRIPS **[25]	NR	NR	NR	NR	NR	Cronbach’s α > 0.82 **(+)**
**DSFS **[26]	NR	NR	NR	NR	NR	NR
	NR	NR	NR	NR	NR	NR
**Balsco index for the assessment of drooling **[1]	NR	NR	NR	NR	NR	NR
**TDS **[27]	NR	NR	NR	yes	Cohen *K* 0,647 **(-)**	NR
**mTDS **[28]	NR	NR	NR	NR	NR	NR
**VAS **[29]	NR	NR	NR	NR	NR	NR
**Modified drooling questionnaire **[30]	ICC 0.86 (95% CI 0.77–0.95, *p* < 0.0001) **(+)** ICC 0.92 (95% CI 0.87–0.97, *p* < 0.0001) **(+)**	Yes	NR	Yes	ICC 0.95 (95% CI 0.914–0.984, *p* < 0.0001) **(+)** ICC 0.96 (95% CI 0.944–0.99, *p* < 0.0001) **(+)**	α Cronbach > 0.867–0.879 **(+)**
**DIS **[31]	NR	NR; yes	NR	Yes	Concordance correlation coefficient 0.85 (standard error 0.05) **(+)**	NR
**DIS-F **[32]	NR	NR	NR	Yes	Concordance correlation coefficient 0.83 (standard error 0.06). Standard error of measurement = 2.6 **(+)**	α Cronbach. = 0.71 **(+)**
**Brazilian Portuguese language version of DIS** [33]	NR	NR	NR	NR	NR	α Cronbach. > 0.72 **(+)**
**Drooling impact questionnaire (short version) **[6, 36]	NR	NR	NR	NR	NR	NR
	NR	NR	NR	NR	NR	NR
**Questionnaire to evaluate impact of drooling on daily living (questionnaire 1 and questionnaire 2) **[8]	NR	NR	NR	NR	NR	NR
**DDISQ **[34]	NR	NR	NR	NR	NR	NR
**Drool rating scale **[35]	NR	NR	NR	NR	NR	NR

*CI* confidence interval, *DDISQ* Daniel Drooling Impact Score Questionnaire, *DIS* Drooling Impact Scale, *DIS-F* French version of Drooling Impact Scale, *DQ* Drooling Quotient, *DQ5* 5-min Drooling Quotient, *DQ5A* 5-min Drooling Quotient during activities, *DQ5R* 5-min Drooling Quotient at rest, *DRIPS* Drooling Infants and Preschoolers Scale, *DSFS* Drooling Severity and Frequency Scale, *ICC* intraclass correlation coefficient, *K* kappa coefficient, *NA* not applicable, *SD* standard deviation, *TDS* Teacher Drooling Scale, *VAS* Visual Analogue Scale, (+) positive rating, (-) negative rating

Among instruments with validity and reliability data, the DQ5 [24] and the modified drooling questionnaire [30] had an overall positive score in terms of quantitative results and methodological quality. Specifically, for the DQ5 [24], most measurement properties in the checklist were rated positively with an overall score of ‘very good’. The 5-min Drooling Quotient during activities (DQ5^A^) was more discriminative for drooling severity than the 5-min Drooling Quotient at rest (DQ5^R^), with a cut-off point of 18 indicating a constant drooling. Criterion validity had been calculated for the DQ5, showing a positive strong correlation between the DQ5 [24] and the DQ [23]. For inter-rater reliability, the DQ5 showed a higher correlation between the scores of the observers.

The modified drooling questionnaire [30] was rated as ‘adequate’ in terms of content validity. Reliability was rated ‘very good’, as it showed a higher correlation between observers’ scores; a cut-off of 24 discriminates between mild and severe drooling.

For the DIS [31], the DIS-F [32], and the Brazilian Portuguese language version of DIS [33], although most items of measurement properties in the checklist were rated positively, the overall score was rated as ‘doubtful’, due to lack of clarity on how missing items were handled. For both TDS [27] and DQ [23], measurement analysis was considered unsatisfactory. The overall score given to the measurement properties tested in the DRIPS [25] ranged from ‘adequate’ to ‘very good’.

The quality scores using ‘worst score counts’ [14] criteria are reported in Table 5. Data on validity and responsiveness of studies are summarised in Table 3; data on reliability are summarised in Table 4.

**Table 5:** Outcome measures, quality appraisal

**Outcome measure**	**Sample size (*n*)**	**COSMIN measurement property **	**COSMIN worst score**	**COSMIN worst score item (s)**
**Bib count **	414 [20]	Criterion validity [20, 38]	Doubtful	Design requirements (unclear whether the criterion can be considered a ‘gold standard’)
	155 [38]			
**Bib weight**	14 [21]	Criterion validity	Doubtful	Design requirements (unclear whether the criterion can be considered a ‘gold standard’; <30 patients in biggest group)
**Sochaniwskyj’s technique **	NR [22]	None	NA	NA
**DQ5 **	162 [24]	Criterion validity	Very good	Design requirements, statistical methods
	"	Reliability	Very good	Design requirements, statistical methods for reliability
	"	Measurement error	Very good	Design requirements, statistical methods for measurement error
**DQ**	14 [23]	Reliability [23]	Inadequate	Design requirements (sample size <30 patients; only one measurement used) and statistical methods (ICC or Pearson or Spearman correlations not calculated)
	155 [38], 113 [30]	Criterion validity [38, 30]	doubtful	Design requirements (unclear whether the criterion can be considered a ‘gold standard’)
**DRIPS**	652 [25]	Content validity	Adequate	Design requirements (not clearly described in all points)
	"	Structural validity	Very good	Statistical methods (confirmatory factor analysis performed; sample size appropriate; clear description of how missing items are handled)
	"	Internal consistency	Very good	Design requirements (evidence that the scale is unidimensional; appropriate sample size; clear description of how missing items are handled) and statistical methods (calculation of Cronbach’s α)
**DSFS Thomas **	36 [26], 155 [38]	Criterion validity [38]	Doubtful	Design requirements (unclear whether the criterion can be considered a ‘gold standard’)
**Balsco index for the assessment of drooling **	NR [1]	None	NA	NA
**TDS**	20 [27]	Criterion validity	Doubtful	Design requirements (unclear whether the criterion can be considered a ‘gold standard’; <30 patients in biggest group)
	"	Reliability	Inadequate	Design requirements (sample size <30 patients)
**mTDS**	39 [28]	None	NA	NA
**VAS **	162 [29]	Criterion validity	Doubtful	Design requirements (unclear whether the criterion can be considered a ‘gold standard’)
**Modified drooling questionnaire **	113 [30]	Content validity	Adequate	Design requirements (not clearly described all points)
	"	Cross-cultural validity	Doubtful	Statistical methods (not clear description of how missing items are handled)
	"	Criterion validity	Doubtful	Design requirements (unclear whether the criterion can be considered a ‘gold standard’)
	"	Reliability	Very good	Design requirements, statistical methods for reliability and measurement error
	"	Internal consistency	Doubtful	Design requirements (not clearly described how missing items are handled)
**DIS **	80 [31]	Content validity	Adequate	Design requirements (not clearly described all points)
	"	Structural validity	Doubtful	Statistical methods (not clearly described how missing items are handled)
	"	Responsiveness	Doubtful	Statistical methods (not clearly described how missing items are handled)
	"	Reliability	Doubtful	Design requirements (sample size of 50–99 patients) and statistical methods (not clearly described how missing items are handled)
	"	Measurement error	Doubtful	Statistical methods (not clearly described how missing items are handled)
**DIS-F**	55 [32]	Cross-cultural validity	Inadequate	Design requirements (sample size of <100 patients)
	"	Responsiveness	Doubtful	Statistical methods (not clearly described how missing items are handled)
	"	Reliability	Doubtful	Statistical methods (not clearly described how missing items are handled)
	"	Measurement error	Doubtful	Statistical methods (not clearly described how missing items are handled)
	"	Internal consistency	Doubtful	Design requirements (not clearly described how missing items are handled)
**Brazilian Portuguese language version of DIS **	40 [33]	Cross-cultural validity	Inadequate	Design requirements (sample size of <100 patients)
	"	Internal consistency	Doubtful	Design requirements (sample size of 30–49 patients; not clearly described how missing items are handled)
**Drooling impact questionnaire (short version) **	45 [36]	Content validity	Adequate	Design requirements (not clearly described in all points)
**Questionnaire to evaluate impact of drooling on daily living (questionnaire 1 and questionnaire 2) **	45 [8]	Content validity	Adequate	Design requirements (not clearly described in all points)
**DDISQ **	414 [34]	Criterion validity	Doubtful	Design requirements (unclear whether the criterion can be considered a ‘gold standard’)
**Drool rating scale **	22 [35]	None	NA	NA

*DDISQ* Daniel Drooling Impact Score Questionnaire, *DIS* Drooling Impact Scale, *DIS-F* French version of Drooling Impact Scale, *DQ* Drooling Quotient, *DQ5* 5-min Drooling Quotient, *DQ5A* 5-min Drooling Quotient during activities, *DQ5R* 5-min Drooling Quotient at rest, *DRIPS* Drooling Infants and Preschoolers Scale, *DSFS* Drooling Severity and Frequency Scale, *NA* not applicable, *TDS* Teacher Drooling Scale, *VAS* Visual Analogue Scale

## Discussion

The paucity of reviews in the medical literature about sialorrhea measurements in children has not allowed a robust use of assessment tools by paediatric experts in disability. Our review has highlighted that although there is a wide range of approaches in the clinical practice to assess children’s saliva management, very few sialorrhea outcome measures are currently available to guide medical decision-making. Clinical evaluation of children with sialorrhea includes a thorough anamnestic collection and physical examination. Paediatric history should focus on age of sialorrhea onset, chronicity, precipitating factors, associated symptoms, developmental history, use of medications as well as family, perinatal history, or past pathologic data. Data acquisition can be expedited by questionnaire administration, resulting in multiple benefits. In fact, this is a reasonable and time-sparing procedure for clinicians to measure sialorrhea severity and its impact on both quality of life and routine daily life. It also allows planning intervention programs and periodically measure outcomes of each intervention. Questionnaire administration can also facilitate a comprehensive evaluation and improve clinician familiarity with sialorrhea assessment. The measures described in this review could be categorised in two main groups: the first aimed at discriminating children depending on severity of sialorrhea and the second aimed not only at evaluating severity, but also sialorrhea impact on children and parents’ lives. Moreover, treatment of sialorrhea can be considered effective not only if its severity decreases, but also if it lessens its impact on the caregiver and improves the child’s quality of life.

Among all assessment instruments that we analysed, only few of them have a description of psychometric properties. Nevertheless, some of the measures reporting their internal attributes can be properly used to assess sialorrhea. Specifically, the DIS [31], the DIS-F [32], and the modified drooling questionnaire [30] can be used as valid and reliable measures of drooling severity and social acceptability in children with developmental disabilities and CP dealing with sialorrhea. Moreover, the DIS [31] and the DIS-F [32] were the only evaluative tools with responsiveness data, being useful for detecting clinically important changes over time. Instead, the modified drooling questionnaire [30] can be used as a discriminative tool, and is also the first questionnaire validated in the Indian paediatric population with CP.

Furthermore, clinicians may undertake an accurate classification of sialorrhea through a quantitative measure: specifically, the physician can objectively assess sialorrhea frequency using the DQ5A in children with developmental disability and moderate-to-profuse sialorrhea [24]. Discriminative properties for the DQ5 in children with infrequent and slight drooling and population groups other than children with developmental disabilities have not been studied yet. Moreover, among questionnaires, the DRIPS [25] can be used by clinicians to monitor sialorrhea, due to the presence of charts created with a reference cohort of children with typical development.

The integration of patient-reported outcomes into clinical care is becoming a standard practice [37]. For children who drool, the subjective opinion of parents provides insight on drooling severity and its relevance, while quantitative methods can help to corroborate subjective findings. For these reasons and as previously reported by van Hulst et al. [24], sialorrhea evaluation should cover quantitative measures and parent or proxy reports in both clinical and research contexts.

## Strengths and limitations

The present review provides insights into the current evidence on the available outcome measures of sialorrhea in children. It also describes important measurement properties that enable dedicated healthcare professionals to choose the best available outcome measure. Strength of this review is the use of a rigorous and stringent methodology. As suggested by the COSMIN checklist [12], the ‘‘worst score counts’’ principle [14] was used to obtain a methodological quality score for each measurement property. A poorer score on any item was considered to represent a fatal flaw. Publication bias is a frequent limitation in most systematic reviews: although many efforts were made to seize all studies, some potentially relevant studies might have been excluded. Specifically, language restriction was an important limitation because it led to the exclusion of a substantial number of potentially relevant studies.

## Future research

Further studies investigating the properties of sialorrhea outcome measures are needed in order to obtain more robust data. Outcome measures should be also evaluated in different population groups. An electronic format of these same tools should be also provided, to obtain real-time data in case face-to-face consultations are not deliverable.

## Conclusions

The measures included in this systematic review varied in the evaluation methods and domains assessed, and measurement properties were often not available. Our findings suggest that a combination of both quantitative measures and parent/proxy questionnaires might provide an adequate measurement of sialorrhea in children. Given the high rates of moderate and severe sialorrhea in different paediatric conditions with disability, the use of valid and reliable measures of sialorrhea might improve physicians’ confidence in its evaluation, support clinical decision-making, enhance efficacy of follow-up after treatments, and optimise research quality.

## Supplementary Information

Below is the link to the electronic supplementary material.

Supplementary file1 (DOCX 14 KB)

Supplementary file2 (DOCX 32 KB)

Supplementary file3 (DOCX 16 KB)

Supplementary file4 (DOCX 19 KB)

## Data Availability

Not applicable.

## Abbreviations

DDISQ: Daniel Drooling Impact Score Questionnaire
DIS: Drooling Impact Scale
DIS-F: French version of Drooling Impact Scale
DQ: Drooling Quotient
DQ5: 5-Minute Drooling Quotient
DQ5^A^: 5-Minute Drooling Quotient during activities
DQ5^R^: 5-Minute Drooling Quotient at rest
DRIPS: Drooling Infants and Preschoolers Scale
DSFS: Drooling Severity and Frequency Scale
TDS: Teacher Drooling Scale
VAS: Visual Analogue Scale
