# Supplementary material for: Drooling outcome measures in paediatric disability: a systematic review
Source: Eur J Pediatr. 2022 Apr 20;181(7):2575–92. doi: 10.1007/s00431-022-04460-5 (PMC9192436; doi:10.1007/s00431-022-04460-5)
Supplement: Supplementary file 1 — Supplementary file1 (DOCX 14 KB) [file 431_2022_4460_MOESM1_ESM.docx]

**Supplementary Table 1.** Methodology of search for articles evaluated in this Review.

| **Database** | **Search strategy** |
| --- | --- |
| **PubMed** | 1. (((child*[MeSH Terms]) OR (infant[Title/Abstract])) OR (pediatrics[MeSH Terms])) OR (adolescent[MeSH Terms]))  2. ((quality of life[MeSH Terms]) OR (questionnaire[MeSH Terms])) OR (assessment, outcomes[MeSH Terms])) OR (test[Title/Abstract]) OR (scale[Title/Abstarct] OR (evaluation[Title/Abstract]) OR (measure [Title/Abstract])  3. ((drooling[MeSH Terms]) OR (sialorrhea[MeSH Terms])  4. (1) AND (2) AND (3)  5. (4) AND Humans |
| **Scopus** | 1. ( TITLE-ABS-KEY ( pediatric ) OR TITLE-ABS-KEY ( child ) OR TITLE-ABS-KEY ( adolescent ) OR TITLE-ABS-KEY ( infant ) )  2. ( TITLE-ABS-KEY ( quality AND of AND life ) OR TITLE-ABS-KEY ( questionnaire ) OR TITLE-ABS-KEY ( assessment ) OR TITLE-ABS-KEY ( scale ) OR TITLE-ABS-KEY ( test ) OR TITLE-ABS-KEY ( evaluation ) OR TITLE-ABS-KEY ( outcome ) OR TITLE-ABS-KEY ( measure ) )  3. ( TITLE-ABS-KEY ( drooling ) OR TITLE-ABS-KEY ( sialorrhea ) )  4. (1) AND (2) AND (3) |
| **Cochrane Library** | 1. ("Child"):ti,ab,kw OR (infant):ti,ab,kw OR (pediatric):ti,ab,kw OR (adolescent):ti,ab,kw  2. (quality of life):ti,ab,kw OR (scale):ti,ab,kw OR (outcome): ti,ab,kw OR (measure):ti,ab,kw OR ("questionaire"):ti,ab,kw AND ("assessment"):ti,ab,kw  3. (“sialorrhea OR drooling”).mp. [mp=ti,ab,kw]  4. (1) AND (2) AND (3) |
| **CINAHL** | 1. AB (children OR adolescents OR youth OR teenager OR pediatric OR paedetric OR kids OR infant)  2. AB (scale OR test OR questionnaire OR assessment OR measure OR inventory OR instrument OR outcome OR quality of life)  3. TI Sialorrhea OR drooling  4. (1) AND (2) AND (3) |
