# Supplementary material for: Drooling outcome measures in paediatric disability: a systematic review
Source: Eur J Pediatr. 2022 Apr 20;181(7):2575–92. doi: 10.1007/s00431-022-04460-5 (PMC9192436; doi:10.1007/s00431-022-04460-5)
Supplement: Supplementary file 3 — Supplementary file3 (DOCX 16 KB) [file 431_2022_4460_MOESM3_ESM.docx]

**Supplementary table 3.** Description of measurement properties [17, 18]

| Property | Description |
| --- | --- |
| Content validity | It is the extent to which the domain of interest is comprehensively sampled by the items in the questionnaire. |
| Criterion validity | It is an estimation of the extent to which a measure agrees with a gold standard.  It is assessed by statistically testing a new measurement technique against an independent criterion or standard (concurrent validity) or against a future standard (predictive validity). |
| Construct validity | It guarantees that measurements resulting from questionnaire responses can be considered and used as a measurement of the phenomenon under study; it is assessed by testing predefined hypotheses; it includes structural validity, hypotheses-testing and cross-cultural validity. |
| Sensitivity and specificity | Sensitivity is the ability to detect most of patients with a disease (few false negatives).  Specificity is the ability to detect people without the disease (few false positives). |
| Reliability | It estimates the stability of measures.  It is the extent to which patients can be distinguished from each other, despite measurement errors (relative measurement error).  It includes intra-rater reliability, inter-rater reliability, test-retest reliability. |
| Internal consistency | It is the extent to which items in a (sub)scale are intercorrelated, thus measuring the same construct. |
| Measurement error | It is the systematic and random error of a patient’s score that is not attributed to true changes in the construct to be measured. |
| Responsiveness | It is the ability of a questionnaire to detect clinically important changes over time. |
