# Supplementary material for: Drooling outcome measures in paediatric disability: a systematic review
Source: Eur J Pediatr. 2022 Apr 20;181(7):2575–92. doi: 10.1007/s00431-022-04460-5 (PMC9192436; doi:10.1007/s00431-022-04460-5)
Supplement: Supplementary file 4 — Supplementary file4 (DOCX 19 KB) [file 431_2022_4460_MOESM4_ESM.docx]

**Supplementary material**

**Description of included tools of sialorrhea**

The following is a brief summary of each outcome measure.

**Bib count** [20]

Bib count is a popular question used by healthcare professionals dealing with sialorrhea and it is recommended as a fundamental element of history-taking. It is based on the number of daily bibs changes.

**Bib weight** [21]

Bib weight is a quantitative assessment method with a definite procedure described by Senner et al. Specifically, prior to the sialorrhea measurement child’s mouth and chin have to be wiped with a paper towel to remove any drool present before the procedure. Next, the subject has to wear a bib for exactly 10 minutes. After the 10-minute period, any saliva on the lips or chin are wiped with the bib. The bib is weighed and the amount of drool produced is determined by subtracting the original bib weight from the weight obtained after the 10- minute period.

**Sochaniwskyj's technique** [22]

In 1982, Sochaniwskyj described a technique based on collection with a chin cup of the saliva leaking from the mouth over a period of 30 minutes. This was repeated five times in order to calculate an average and thus avoid variability of the data obtained. The technique did not involve the collection of whole saliva, but only the saliva leaking through the lips and reaching the chin.

**5-minute Drooling Quotient (DQ5)** [24]

The Drooling Quotient was originally adopted by Rapp [23] and modified afterwards. It represents a semiquantitative, direct observational method that evaluates sialorrhea by measuring leaked saliva from the mouth (so-called anterior drooling). In its original form, Rapp used a ‘teacher prompt device’ to obtain measurements of sialorrhea in moment-by-moment sampling observations in the classroom. Hulst et al [24] modified the DQ in the 5-minute Drooling Quotient (DQ5). The DQ5 requires the evaluation of sialorrhea in two different conditions too: “rest” and “activity”. During 5 minutes, for every interval of 15 seconds (20 intervals), the presence or absence of sialorrhea is determined. The value of the DQ is obtained from the ratio of the number of episodes of sialorrhea to the number of observations. Hults et al suggest the use of a cut-off point to guide clinical decision-making. A drooling quotient of 18 or higher means that the sialorrhea problem is at least frequent and/or has not been satisfactorily resolved by previous treatment. This cut-off point may be useful in deciding whether intervention is needed.

**Drooling Infants and Preschoolers Scale (DRIPS)** [25]

Van Hulst et al. developed a 20 items parent-reported questionnaire to quantify sialorrhea severity in young typically developed children during meaningful daily activities. Authors also developed sex-specific percentile curves for sialorrhea in four situations for children in different age groups, that allow health professionals to monitor the developmental stages of sialorrhea behaviour up to the age of 4 years and timely initiate individually targeted interventions. Authors suggest to use the 97^th^ percentile as the outer most percentile cut-off value indicating pathological drooling. Children with DRIPS scores above the 85^th^ percentile could be considered as ‘at risk’.

**Drooling Severity and Frequency Scale (DSFS)** [26]

The Drooling Severity and Frequency Score (DSFS) is a subjective method whereby parents are asked to rate the severity and frequency of sialorrhea of their child. The score is the sum of the severity (5 point) and frequency (4 point) sub-scores. Severity of sialorrhea ranges from 1 (dry: never drools), 2 (mild, only the lips are wet), 3 (moderate, wet on the lips and chin), 4 (severe: drools to the extent that clothing becomes damp) to 5 (profuse drooling: clothing, hands, tray and objects become wet). Frequency of sialorrhea ranges from 1 (never drools), 2 (occasional drooling), 3 (frequent drooling) to 4 (constant drooling). The scale was created by Thomas-Stonell and Greenberg in the 1988 [26], and its validity was studied by Rashnoo et al in 2012 [38]. Mato et al. used the modified Thomas-Stonell and Greenberg visual scale simplified into three grades: 1 = dry; 2 = mild/moderate; 3 = severe/fulsome [40].

**Blasco index for the assessment of drooling** [1]

The categorization of Blasco et al. (1992) quantifies sialorrhea on a scale of 0 to 3: (0) absent (no evidence of drooling of drooling of saliva); (1) mild drooling (saliva spills onto the lips but not beyond the vermillion border); (2) moderate drooling (saliva reaches chin); (3) severe drooling (characterized by dripping of saliva onto clothing).

**Teacher Drool Scale (TDS)** [27]

The TDS was created by Camp-Bruno in 1989. It was originally administered by teachers and classroom staff who were asked to observe and rate sialorrhea over a full school-day of 20 selected students with CP. The TDS comprises a 5-point scale to define the degree of sialorrhea: 1. No drooling; 2. Infrequent drooling, small amount; 3. Occasional drooling, on and off all day; 4. Frequent drooling, but not profuse; 5. Constant drooling, always wet.

**Modified Teacher Drool Scale** [28]

This 9-point scale was created by Mier in 2000 and measures frequency and level of sialorrhea, where 1. “Dry, never drools”, 2. “Mild; only the lips are wet occasionally”, 3. “Mild; only the lips are wet, but frequently”, 4. “Moderate; wet on the lips and chin occasionally”, 5. “Moderate; wet on the lips and chin frequently”, 6. “Severe; drools to the extent that clothing becomes damp occasionally” 7. “Severe; drools to the extent that clothing becomes damp frequently” 8.” Profuse; clothing, hands, and objects become wet occasionally”, 9. “Profuse; clothing, hands, and objects become wet frequently”.

**VAS [29]**

The visual analogue scale (VAS) consists of a horizontal line, without visible subdivisions, 10 cm in length used to rate severity of sialorrhea. Parents are asked to indicate the average sialorrhea severity, considering that the left end of the scale means no drooling and the right end means severe drooling. VAS is included in some of the drooling questionnaire, i.e. Drooling impact questionnaire (short version) [6], but it also have been used singly to investigate sialorrhea therapy results after regular time intervals i.e. in the Jongerius et al. study [29].

**Modified drooling questionnaire** [30]

The Modified drooling questionnaire was developed by Job et al in 2018 in order to assess severity of sialorrhea and its social impact in children with CP in the Indian scenario. Authors found that a score of 24 is the most sensitive and specific point to discriminate between the mild and severe droolers in children with CP.

**Drooling Impact Scale (DIS)** [31]

The Drooling Impact Scale was designed to evaluate longitudinal changes of sialorrhea impact in children with developmental disabilities and cerebral palsy. The 10 items (scored on a 10-point scale) were specifically generated to quantify the short- to medium-term treatment benefits of saliva-control interventions (botulinum toxin injections or drug medication). The scale can be filled-out by the child’s parents or caregivers and to minimize the short-term variability of sialorrhea, the evaluation takes place over an entire week. A 10-point reduction is considered significant. The scale has been translated and validated in French [32] and Brazilian-Portuguese [33].

**Drooling impact questionnaire (short version)** [6]

The Drooling impact questionnaire is a short version (17 items) of two questionnaire developed by van der Burg et al in 2006 [8]. To enhance the applicability, authors eliminated questions that were not distinctive in the evaluation of the effect of reduced sialorrhea after botulinum toxic injections. This questionnaire was designed to evaluate sialorrhea severity in specific daily life situations and its economic consequences, sialorrhea impact on daily care, social interaction and emotional development (self-esteem). The questionnaire consists of multiple-choice as well as open-ended questions and several Visual Analogue Scales (VAS). The Kok et al. study [36] was the first to have an appendix in which this questionnaire was published in a peer-reviewed journal.

**Questionnaire to evaluate impact of drooling on daily living (Questionnaire 1; Questionnaire 2)** [8]

The two questionnaire were developed by van der Burg 2006 especially for their study, in order to investigate the impact of sialorrhea on daily life before and after treatment (scopolamine and botulinum toxin) in children with CP and severe sialorrhea. In the first questionnaire questions are aimed at assessing sialorrhea severity in specific daily situations, the practical consequences for daily care that may be influenced by sialorrhea itself, or, possibly, could improve after treatments. In the second questionnaire questions are focused on the impact of sialorrhea on social interaction and on emotional development (self-esteem).

**Daniel Drooling Impact Score Questionnaire (DDISQ)** [34]

The Daniel Drooling Impact Score Questionnaire (DDISQ) is a comprehensive validated 20-item questionnaire that assesses sialorrhea severity and its burden on the child and family. The DDISQ is sub-divided into 3 categories: quantity, impact on caregiver/family, and impact on child. Each of the 20 items assessed has a 5-point Likert scale filled by the caregiver.

**Drool rating scale** [35]

The drool rating scale was created in 2002 by Suskind et al. in order to assess efficacy of intraglandular BTX-A in the treatment of sialorrhea in children with CP. The questionnaire has to be completed by the caregiver of children and consists of 14 multiple-choice questions related to physical, quality of life and caregiver issues that may have changed with alteration in salivation.
